# Long-term outcomes in patients with polyarticular juvenile idiopathic arthritis receiving adalimumab with or without methotrexate

**DOI:** 10.1136/rmdopen-2020-001208

**Published:** 2020-07-13

**Authors:** Daniel J Lovell, Hermine I Brunner, Andreas O Reiff, Lawrence Jung, Katerina Jarosova, Dana Němcová, Richard Mouy, Christy Sandborg, John F Bohnsack, Dirk Elewaut, Christos Gabriel, Gloria Higgins, Isabelle Kone-Paut, Olcay Y Jones, Veronika Vargová, Elizabeth Chalom, Carine Wouters, Ivan Lagunes, Yanna Song, Alberto Martini, Nicolino Ruperto, for the Pediatric Rheumatology Collaborative Study Group (PRCSG) and the Paediatric Rheumatology International Trials Organization (PRINTO)

**Affiliations:** 1 Department of Pediatrics, Division of Rheumatology, University of Cincinnati, Cincinnati Children’s Hospital Medical Center, PRCSG Coordinating Center, Cincinnati, Ohio, USA; 2 Department of Pediatrics, University of Southern California Keck School of Medicine, Los Angeles, California, USA; 3 Division of Rheumatology, Children’s Hospital of Los Angeles, Los Angeles, California, USA; 4 Department of Rheumatology, Children’s National Medical Center for Cancer and Immunology Research, Washington, District of Columbia, USA; 5 Institute of Rheumatology, Prague, Czech Republic; 6 Department of Rheumatology, First Faculty of Medicine, Charles University, Prague, Czech Republic; 7 Department of Pediatrics and Adolescent Medicine, First Faculty of Medicine, Charles University and General University Hospital, Prague, Czech Republic; 8 Pediatric Rheumatology, Univeristé Paris-Descartes and Hôpital Necker-Enfants Malades, Paris, France; 9 Pediatric Rheumatology, Lucile Packard Children’s Hospital at Stanford, Palo Alto, California, USA; 10 Department of Pediatrics, Division of Allergy, Immunology and Pediatric Rheumatology, University of Utah, Salt Lake City, Utah, USA; 11 Rheumatology, University Hospital Gent, Gent, Belgium; 12 VIB Center for Inflammation Research, Gent University, Gent, Belgium; 13 Pediatric Rheumatology, Children’s Hospital of the King’s Daughters, Norfolk, Virginia, USA; 14 Department of Pediatrics, The Ohio State University College of Medicine, Columbus, Ohio, USA; 15 Department of Paediatric Rheumatology and CEREMAI, Hôpital De Bicêtre, National Reference Centre for Auto-inflammatory Diseases, Le Kremlin-Bicêtre, Paris, France; 16 Pediatric Rheumatology, Walter Reed National Military Medical Center, Bethesda, Maryland, USA; 17 Pediatric Rheumatology Unit, Faculty Hospital, Kosice, Slovakia; 18 Pediatric-Rheumatology, Saint Barnabas Medical Center, Livingston, New Jersey, USA; 19 Pediatric Immunology, University Hospital Gasthuisberg, Leuven, Belgium; 20 AbbVie Inc, North Chicago, Illinois, USA; 21 Dipartimento di Neuroscienze, Riabilitazione, Oftalmologia, Genetica e Scienze Materno-Infantili (DiNOGMI),, Università degli Studi di Genova, Genoa, Italy; 22 Clinica Pediatrica e Reumatologia—PRINTO, IRCCS Istituto Giannina Gaslini, Genova, Italy

**Keywords:** Juvenile Idiopathic Arthritis, Anti-TNF, Adult Onset Still’s Disease, Methotrexate, Arthritis, Dermatomyositis, Lupus Erythematosus, Systemic, Familial Mediterranean Fever, Autoimmune Diseases

## Abstract

**Objectives:**

Long-term safety and efficacy of adalimumab among patients with juvenile idiopathic arthritis (JIA) was evaluated through 6 years of treatment.

**Methods:**

Children aged 4–17 years with polyarticular JIA were enrolled in a phase III, randomised-withdrawal, double-blind, placebo-controlled trial consisting of a 16-week open-label lead-in period, 32-week randomised double-blind period and 360-week long-term extension. Patients were stratified by baseline methotrexate use. Adverse events (AEs) were monitored, and efficacy assessments included JIA American College of Rheumatology (JIA ACR) 30%, 50%, 70% or 90% responses and the proportions of patients achieving 27-joint Juvenile Arthritis Disease Activity Score (JADAS27) low disease activity (LDA, ≤3.8) and inactive disease (ID, ≤1).

**Results:**

Of 171 patients enrolled, 62 (36%) completed the long-term extension. Twelve serious infections in 11 patients were reported through 592.8 patient-years of exposure. No cases of congestive heart failure-related AEs, demyelinating disease, lupus-like syndrome, malignancies, tuberculosis or deaths were reported. JIA ACR 30/50/70/90 responses and JADAS27 LDA were achieved in 66% to 96% of patients at week 104, and 63 (37%) patients achieved clinical remission (JADAS27 ID sustained for ≥6 continuous months) during the study. Attainment of JIA ACR 50 or higher and JADAS27 LDA or ID in the initial weeks were the best predictors of clinical remission. Mean JADAS27 decreased from baseline, 22.5 (n=170), to 2.5 (n=30) at week 312 (observed analysis).

**Conclusions:**

Through 6 years of exposure, adalimumab was well tolerated with significant clinical response (up to clinical remission) and a relatively low retention rate.

## INTRODUCTION

Juvenile idiopathic arthritis (JIA) describes a clinically heterogeneous group of arthritides of unknown cause that begin before 16 years of age and often continue into adulthood.^1–^^3^ The American College of Rheumatology (ACR) recommendations suggest initiating treatment with non-steroidal anti-inflammatory drugs (NSAIDs) or methotrexate or, in more severe cases, biologics.^4^ Because use of a particular biological agent in an individual patient may be limited by long-term toxicity, inadequate response or the inability to maintain an acceptable level of disease control, it is important to have knowledge and regulatory approval of several biological therapies for the treatment of JIA, including tumour necrosis factor inhibitors (such as etanercept, adalimumab, infliximab and golimumab) or biologics with other mechanisms of action such as abatacept, tocilizumab and canakinumab.^4–^^11^

Adalimumab has a favourable benefit–risk profile in children with JIA.^1 6 12–^^14^ A long-term, 4-period, phase III study of up to 360 weeks of adalimumab treatment with or without methotrexate (ClinicalTrials.gov Identifier: NCT00048542) was conducted in children with JIA who had active disease and had failed NSAIDs.^6^ The results from the double-blind, placebo-controlled period demonstrated that in patients who initially responded to open-label adalimumab treatment, significantly fewer disease flares occurred in patients continuing to receive adalimumab compared with those who switched to placebo; treatment response was sustained during an additional 104 weeks of open-label adalimumab treatment.

The objective of this follow-up analysis was to investigate the long-term safety and efficacy of adalimumab with or without methotrexate through up to 6 years of exposure and to assess predictors of sustained disease control in patients with polyarticular JIA through up to 6 years of treatment.

## METHODS

### Study design and patients

The methods and results of the first 48 weeks of this study have been previously published.^6^ Briefly, this was a phase III, 4-period study that consisted of a 16-week open-label adalimumab lead-in period followed by a 32-week randomised, withdrawal, double-blind, placebo-controlled period and a 2-part, long-term extension for up to 6.9 years (360 weeks; online supplementary figure 1). Adalimumab was administered based on body surface area (BSA; 24 mg/m^2^, maximum of 40 mg every other week) during the open-label lead-in and double-blind periods. Patients with ≥30% improvement in the JIA ACR 30 response at the end of the open-label lead-in period were eligible for randomisation for the double-blind period. Patients completing the double-blind period were eligible to enter into the 2-part long-term extension in which patients received open-label adalimumab based on BSA (24 mg/m^2^, maximum of 40 mg every other week) for up to 44–136 weeks, and then fixed-dose (FD) adalimumab (<30 kg, 20 mg every other week; ≥30 kg, 40 mg every other week) for up to 224 weeks.

**Figure 1:**
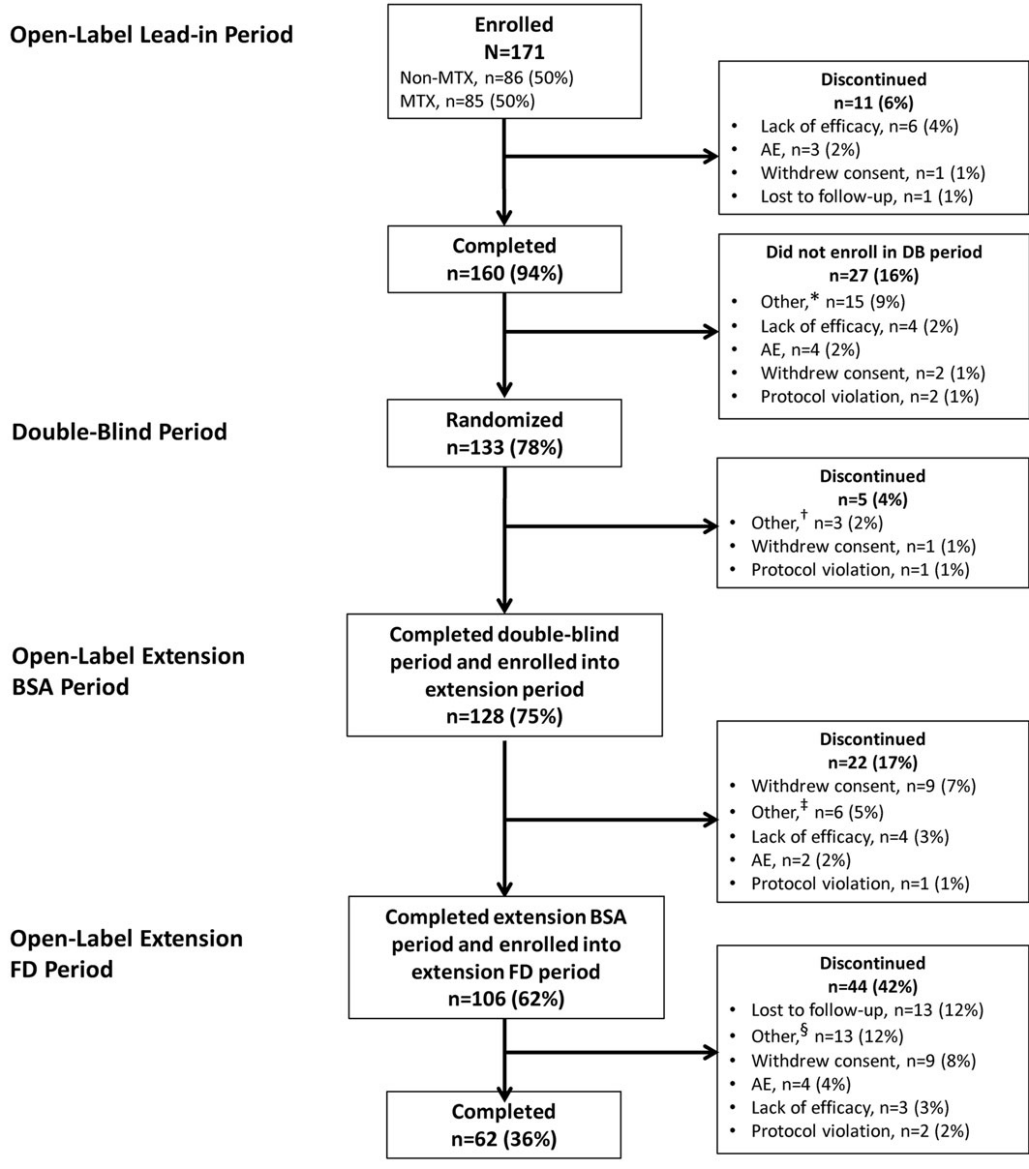
Patient disposition. *Not randomised at week 16 (n=12), sponsor decision (n=2), non-responder at week 16 (n=1). ^†^Sponsor request/decision (n=2), randomised in error (n=1). ^‡^Site not participating in the extension (n=5), remission (n=1). ^§^Not known (n=13). AE, adverse event; BSA, body surface area; DB, double-blind; FD, fixed- dose; MTX, methotrexate.

**Figure 2:**
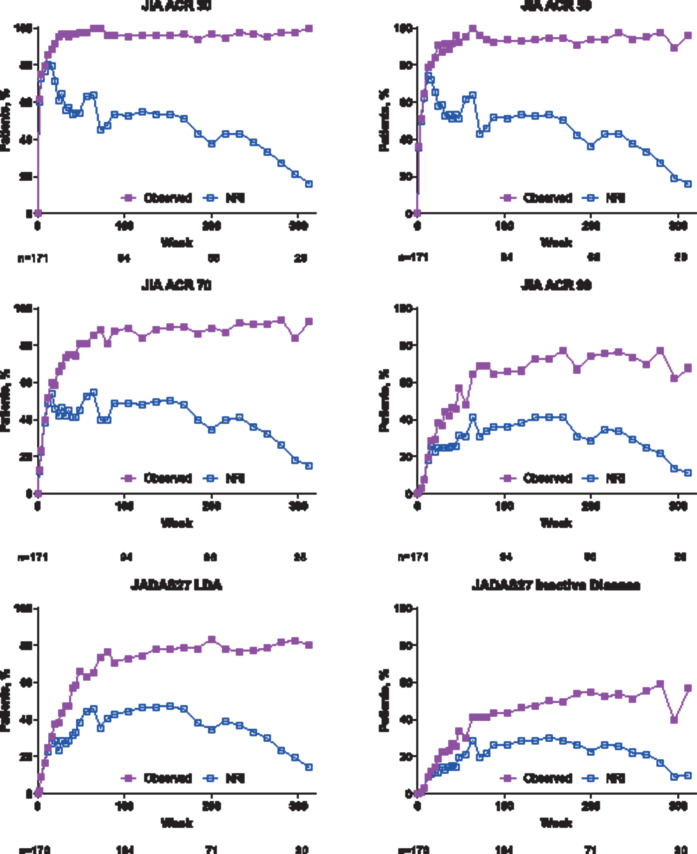
Percentage of patients achieving JIA ACR 30/50/70/90, JADAS27 LDA and JADAS27 inactive disease over time. (A) Observed and (B) NRI analyses. JADAS27 LDA, ≤3.8; JADAS27 inactive disease, ≤1. For observed analysis, n values are shown for weeks 0, 104, 200 and 312; for NRI analysis, N=171. JADAS27, 27-joint Juvenile Arthritis Disease Activity Score; JIA ACR 30/50/70/90, 30%, 50%, 70%, or 90% improvement in the Juvenile Idiopathic Arthritis American College of Rheumatology response; LDA, low disease activity; NRI, non-responder imputation.

10.1136/rmdopen-2020-001208.supp1 Supplementary data

10.1136/rmdopen-2020-001208.supp2 Supplementary data

10.1136/rmdopen-2020-001208.supp3 Supplementary data

10.1136/rmdopen-2020-001208.supp4 Supplementary data

10.1136/rmdopen-2020-001208.supp5 Supplementary data

10.1136/rmdopen-2020-001208.supp6 Supplementary data

10.1136/rmdopen-2020-001208.supp7 Supplementary data

10.1136/rmdopen-2020-001208.supp8 Supplementary data

10.1136/rmdopen-2020-001208.supp9 Supplementary data

10.1136/rmdopen-2020-001208.supp10 Supplementary data

10.1136/rmdopen-2020-001208.supp11 Supplementary data

10.1136/rmdopen-2020-001208.supp12 Supplementary data

10.1136/rmdopen-2020-001208.supp13 Supplementary data

10.1136/rmdopen-2020-001208.supp14 Supplementary data

10.1136/rmdopen-2020-001208.supp15 Supplementary data

10.1136/rmdopen-2020-001208.supp16 Supplementary data

10.1136/rmdopen-2020-001208.supp17 Supplementary data

10.1136/rmdopen-2020-001208.supp18 Supplementary data

10.1136/rmdopen-2020-001208.supp19 Supplementary data

10.1136/rmdopen-2020-001208.supp20 Supplementary data

10.1136/rmdopen-2020-001208.supp21 Supplementary data

10.1136/rmdopen-2020-001208.supp22 Supplementary data

### Participants

Children aged 4–17 years with polyarticular-course juvenile rheumatoid arthritis (JRA) from any of the three JRA onset subtypes (systemic, oligoarticular, polyarticular) were eligible. This would correspond to the JIA eligible categories of systemic, extended oligoarthritis and polyarticular rheumatoid factor (RF positive and negative). In addition, eligibility criteria included patients with active disease (at least five active joints plus at least three joints with limitation of motion) who had not responded adequately or tolerated treatment with NSAIDs or methotrexate.^6^ Patients were excluded if they had previously received treatment with other biological agents at any time or were recently treated with intravenous immunoglobulin, cytotoxic agents, investigational agents, corticosteroids or disease-modifying antirheumatic drugs (DMARDs) other than methotrexate.

Children were enrolled from 31 centres in 8 countries, belonging to the Pediatric Rheumatology Collaborative Study Group^15^ and the Pediatric Rheumatology International Trials Organization (PRINTO).^16^

Patients were stratified by baseline methotrexate use at the beginning of the open-label lead-in period. Patients in the non-methotrexate stratum either had never received methotrexate or had discontinued methotrexate at least 2 weeks before administration of the study drug. Patients in the methotrexate stratum had received methotrexate at a stable dosage of at least 10 mg/m^2^ per week for the 3-month period before screening and continued to receive methotrexate at the same dosage during the open-label lead-in and double-blind periods. During the extension periods, patients could change their methotrexate dosing regimen.

### Efficacy and safety outcomes

Safety was assessed by monitoring adverse events (AEs), laboratory values, physical examination and vital signs throughout the study and 70 days beyond the last dose of adalimumab. Serious AEs were defined as events that were fatal or life-threatening, required hospitalisation or prolonged hospitalisation, resulted in congenital anomaly or persistent or significant disability/incapacity, required medical or surgical intervention to prevent a serious outcome, or other medically important conditions (eg, miscarriage/spontaneous abortion, elective abortion). AEs were coded using the Medical Dictionary for Regulatory Activities version 12.1. Anti-adalimumab antibodies (AAAs) were also assessed during the open-label lead-in and double-blind periods; two AAA assessments were done during the open-label extension at weeks 12 and 16.

Long-term efficacy assessments over time included the proportion of patients achieving JIA ACR 30/50/70/90 response criteria defined as ≥30%, ≥50%, ≥70% or ≥90% improvement from baseline in three of six JIA core set variables and a worsening of >30% from baseline in no more than one of the JIA core set variables.^17^ The variables included Physician Global Assessment of disease activity (10 cm visual analogue scale (VAS); 0=inactive; 10=maximum activity), Patient or Parent Global Assessment of overall well-being (10 cm VAS; 0=very good; 10=very poor), number of active joints (joints with swelling not due to deformity or joints without swelling but with limitation of passive movement, and with pain, tenderness or both), number of joints with limitation of passive movement, physical function using the Childhood Health Assessment Questionnaire Disability Index (CHAQ-DI; range, 0–3; 0=no disability and 3=severe disability)^18 19^ and C reactive protein (CRP) as a measure of inflammation.

Other assessed endpoints included Patient or Parent Global Assessment of Pain (within last week; 10 cm VAS; 0=no pain; 10=severe pain), the proportion of patients achieving 27-joint Juvenile Arthritis Disease Activity Score (JADAS27) low disease activity (LDA, ≤3.8) and inactive disease (ID, ≤1),^20^ mean change from baseline in JADAS27 and CHAQ-DI, the proportion of patients achieving JADAS27 clinical remission (defined as JADAS27 ID sustained for ≥6 continuous months)^20^ and time to first JADAS27 ID. JADAS27 consists of Physician Global Assessment of overall disease activity (10 cm VAS), Patient or Parent Global Assessment of overall well-being (10 cm VAS), number of active joints assessed in 27 joints and erythrocyte sedimentation rate (normalised to a 0–10 scale). JADAS27 is calculated as the sum of the scores, yielding a score of 0–57.^20^

### Predictor analyses

Predictor and regression tree analyses were used to identify baseline and postbaseline factors associated with the achievement of JADAS27 clinical remission status. Tested baseline variables were sex, race, weight, CRP, CHAQ-DI, Physician Global Assessment of disease activity, Patient or Parent Global Assessment of pain, Patient or Parent Global Assessment of overall well-being, RF (positive vs negative), swollen joint count, active joint count, tender joint count, pain of passive movement joint count, limitation of passive movement joint count, prior DMARD use, concurrent methotrexate use and duration of JIA at baseline. Postbaseline variables were JIA ACR 30/70/ 90 responses, JADAS27 LDA status and JADAS27 ID at weeks 4, 8, 12 and 16.

### Statistical analysis

Efficacy analyses were performed in the intention-to-treat population (any patient who received ≥1 dose of study drug). Data were assessed as observed without imputation (in patients with non-missing responses at the assessed visit; patients who discontinued from the study were considered as non-responders from that point on) and using non-responder imputation (NRI; for patients who did not enrol into double-blind trial, discontinued from the study or had missing data for any reason) as well as last observation carried forward (LOCF). A subgroup analysis was performed stratifying patients into those using concomitant methotrexate and those not using methotrexate. The safety population consisted of all patients exposed to adalimumab at any point during the study. Rates of AEs are reported as events per 100 patient-years (PY) of exposure. For the predictor analysis, HR and 95% CI are reported. Univariate Cox regression analysis model with each variable as an independent variable was fit; p values at <0.05 level were considered statistically significant.

### Patient and public involvement

This research was done without any formal patient/patient organisation involvement in study design, development of patient-relevant outcomes, interpretation of results, or the writing or editing of the manuscript.

## RESULTS

### Patients and baseline characteristics

A total of 171 patients were enrolled in the open-label period; of these, 133 were randomised to receive adalimumab or placebo during the double-blind period (figure 1). Overall, 128 (75%) patients completed the double-blind period and entered the long-term extension, with 62 (36%) patients completing it. Primary reasons for study discontinuation were withdrawal of consent (n=20), lost to follow-up (n=14) and ‘other’ reasons (n=22; figure 1). The mean age at baseline was 11.3 years, 79% of patients were female, mean disease duration was 3.8 years and mean JADAS27 score was 22.5 (table 1). The 128 patients who entered the long-term extension had similar baseline characteristics as the overall population.

**Table 1:** Patient demographics and baseline disease characteristics

Characteristic*	Patients (N=171)	Patients entered in long-term extension (n=128)
Age, years	11.3 (3.5)	11.3 (3.6)
Disease duration, years	3.8 (3.9)†	3.7 (3.6)‡
Female, n (%)	135 (78.9)	98 (76.6)
White, n§ (%)	157 (95.2)	121 (96.0)
Body weight, kg	42.2 (18.8)	43.5 (19.5)
Rheumatoid factor positive, n¶ (%)	37 (22.0)	27 (21.6)
Prior DMARDs not including methotrexate, n (%)	44 (25.7)	36 (28.1)
Prior methotrexate use, n (%)	103 (60.2)	83 (64.8)
Tender joint count	11.4 (12.4)	12.0 (12.8)
Swollen joint count	14.8 (9.4)	14.9 (9.1)
Pain on passive motion joint count	10.3 (11.7)	9.5 (10.4)
Limitation of passive motion joint count	13.5 (9.2)	13.4 (9.4)
Active joint count	17.2 (10.4)	17.2 (10.3)
CRP, mg/dL	2.6 (4.1)§	2.4 (4.1)¶
Elevated CRP||, n (%)	112 (65.9)§	79 (62.2)¶
Physician Global Assessment of disease activity#	5.9 (1.8)	5.8 (1.8)
Patient or Parent Global Assessment of overall well-being**	4.8 (2.3)§	4.8 (2.3)¶
Patient or Parent Global Assessment of pain††	5.0 (2.5)	4.9 (2.5)
Childhood Health Assessment Questionnaire Disability Index‡‡	1.1 (0.7)	1.0 (0.7)

Tender joint count, pain on passive motion joint count and limitation of passive motion joint count based on 75 joints; swollen joint count based on 66 joints.

*Data are mean (SD) unless otherwise noted.

†n=170.

‡n=127.

§n=165 and n=126.

¶n=168 and n=125.

||Elevated CRP defined as >0.0287 mg/dL.

#0–10 cm visual analogue scale (0=inactive; 10=maximum activity).

**0–10 cm visual analogue scale (0=very good; 10=very poor).

††0–10 cm visual analogue scale (0=no pain; 10=very severe pain).

‡‡Mean rating of 8 category scores (0=no difficulty to perform activity; 3=complete inability to perform activity).

CRP, C reactive protein; DMARDs, disease-modifying antirheumatic drugs.

### Safety

A total of 3605 (608.1/100 PY) AEs and 75 (12.7/100 PY) serious AEs were reported through 592.8 PY of adalimumab exposure. The incidence of AEs and serious AEs possibly related to the study drug were 1394 (235.2/100 PY) and 19 (3.2/100 PY), respectively (table 2). Injection site reactions (912 (153.8/100 PY)) and infections (880 (148.4/100 PY)) were the most common AEs. Twelve serious infections in 11 (6%) patients were reported (3 events of appendicitis, 2 events of herpes zoster and 1 event each of bronchopneumonia, cervicitis, genital herpes, pharyngitis, pneumonia, urinary tract infection and viral infection); of these, 7 were categorised by the investigator as possibly or probably related to the study drug (3 (viral infection, herpes zoster, pharyngitis) occurred during adalimumab plus methotrexate regimen and 4 (genital herpes, pneumonia, herpes zoster, bronchopneumonia) during or after adalimumab regimen). No cases of congestive heart failure-related AEs, demyelinating disease, lupus-like syndrome, malignancies, tuberculosis or deaths were reported during the study. Twenty patients (3.4/100 PY) discontinued from the study owing to AEs (table 2). Injection site pain (28%), injection site reaction (16%), headache (9%) and upper respiratory tract infection (9%) were the most common AEs reported (online supplementary table 1).

**Table 2:** Rates of adverse events

AE, Events (events per 100 PY of follow-up)	Patients (N=171) 592.8 PY
Any AE	3605 (608.1)
AE possibly drug related	1394 (235.2)
Serious AE	75 (12.7)
Serious AE possibly drug related	19 (3.2)
Severe AE	52 (8.8)
AE leading to discontinuation	20 (3.4)
Infection	880 (148.4)
Serious infection*	12 (2.0)
Opportunistic infection†	2 (0.3)
Tuberculosis	0
Malignancy	0
Injection site reaction	912 (153.8)
Death	0

*Twelve serious infections were reported in 11 patients: 3 events of appendicitis, 2 events of herpes zoster and 1 event each of bronchopneumonia, cervicitis, genital herpes, pharyngitis, pneumonia, urinary tract infection and viral infection.

†One event of oral candidiasis and one event of cytomegalovirus infection.

AE, adverse event; PY, patient-years.

Incidence of AEs was similar among patients receiving and not receiving concomitant methotrexate with the exception of AEs leading to discontinuation (methotrexate use, n=2 (2%) vs non-methotrexate use, n=7 (8%)) and a few individual AEs, eg, worsening of juvenile arthritis and oropharyngeal pain (online supplementary table 1).

The rate of serious AEs, severe AEs, AEs leading to discontinuation of study drug and serious infectious AEs were <10% in both AAA-negative and -positive groups during the open-label lead-in period and double-blind period (online supplementary tables 2 and 3). The rates of AEs leading to discontinuation of study drug and infectious AEs were lower in the AAA-positive groups than in the AAA-negative groups. Injection site reactions reported as AEs were similar among the two groups. Overall, no increased safety risk was observed in patients who were AAA positive versus those who were AAA negative in the presence or absence of methotrexate. Because AAAs were not assessed beyond week 16 of the open-label extension, no analysis could be done past the double-blind period.

### Efficacy

JIA ACR 30 was achieved as early as week 2 of the lead-in period (103/167 patients–observed analysis: 62%; NRI analysis: 60%). In the observed analysis, the majority of patients had achieved JIA ACR 30 (90/94 (96%)), JIA ACR 50 (88/94 (94%)), JIA ACR 70 (84/94 (89%)) and JIA ACR 90 (62/94 (66%)) responses (NRI analysis: 36% to 53%) at week 104 (year 2; figure 2).

Similarly, the majority of patients achieved JADAS27 LDA (observed analysis: 73%; NRI analysis: 44%) at week 104 (year 2); JADAS27 ID was achieved by 43% of patients in the observed analysis and 26% in the NRI analysis at week 104 (figure 2). The response rates were generally maintained through week 312 (year 6; figure 2).

When stratified by concomitant methotrexate use, there was a trend for numerically higher JIA ACR 30/50/70/90 response (online supplementary figure 2) and higher rate of JADAS27 LDA and JADAS27 ID (online supplementary figure 3A) among patients receiving concomitant methotrexate versus those not receiving methotrexate in the NRI analysis but not in the observed analysis.

**Figure 3:**
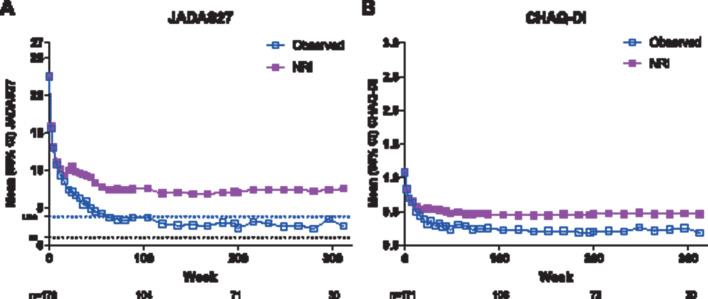
Mean (A) JADAS27 and (B) CHAQ-DI over time, observed and LOCF analyses. N values for observed analysis are shown for weeks 0, 104, 200 and 312. Dotted lines represent JADAS27 LDA (≤3.8) and JADAS ID (≤1) cut-offs. CHAQ-DI, Childhood Health Assessment Questionnaire Disability Index; ID, inactive disease; JADAS27, 27-joint Juvenile Arthritis Disease Activity Score; LDA, low disease activity; LOCF, last observation carried forward; NRI, non-responder imputation.

**Figure 4:**
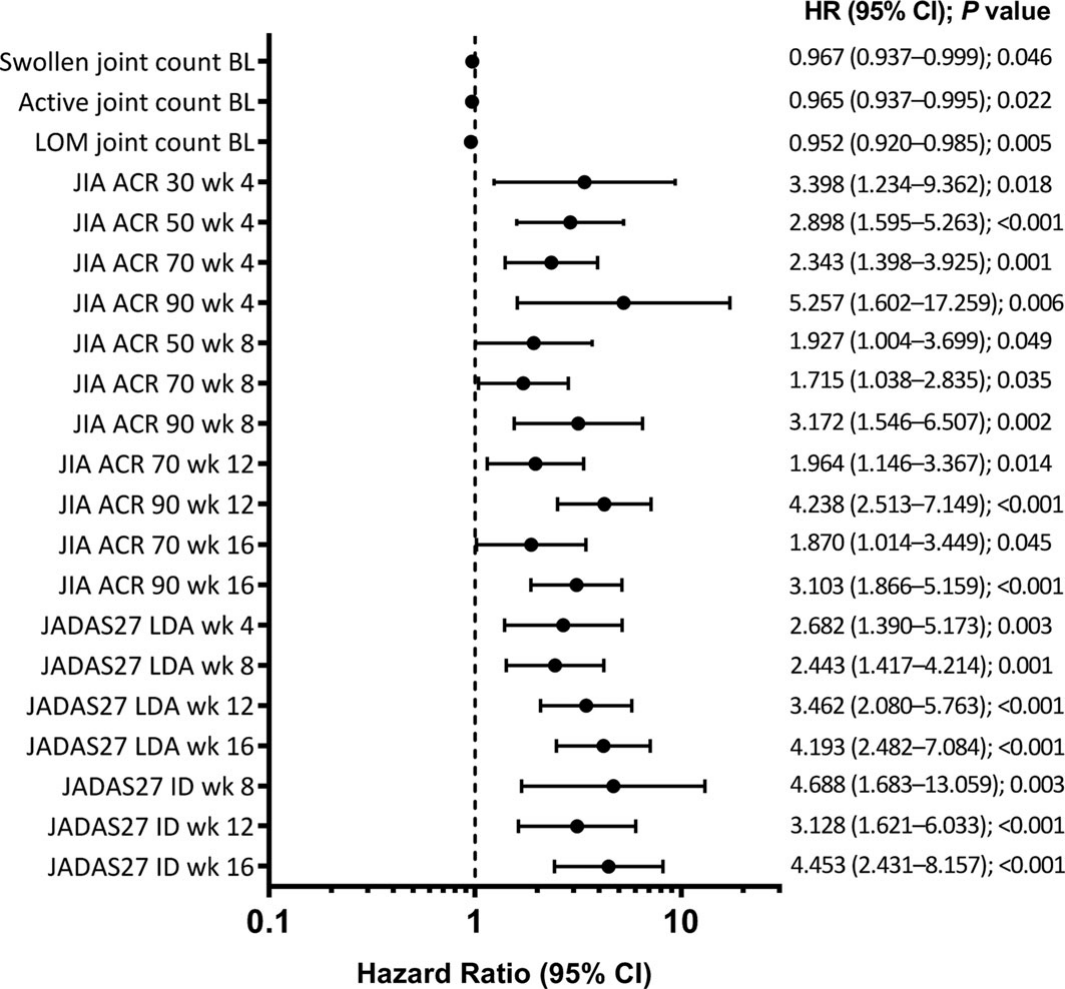
Baseline and postbaseline (JIA ACR responses at weeks 4, 8, 12 and 16) predictors of JADAS27 clinical remission (JADAS27 ≤1 sustained for ≥6 continuous months). BL, baseline; ID, inactive disease; JADAS27, 27-joint Juvenile Arthritis Disease Activity Score; JIA ACR 30/50/70/90, 30%, 50%, 70% or 90% improvement in the Juvenile Idiopathic Arthritis American College of Rheumatology response; LDA, low disease activity; LOM, limitation of movement; wk, week.

JIA improved during the study as assessed by a decrease in mean JADAS27 score of 22.5 (n=170) at baseline to 2.5 (n=30; mean reduction of −16.7 (n=29)) by week 312 in observed analysis and to 7.5 (mean reduction of −14.9) in LOCF analysis (figure 3). A total of 63 (37%) patients achieved JADAS27 clinical remission (ie, sustained JADAS27 ID ≥6 continuous months) during the study; the median time to reach JADAS27 clinical remission was 216 weeks (4.2 years). Of the 106 patients completing the BSA dosing period and entering the FD dosing period of the extension, adalimumab dose was increased in half (n=53) and remained the same (n=50) or was decreased (n=3) in the other half. JIA ACR response rates were maintained during the extension periods, regardless of dose change.

When stratified by concomitant methotrexate use, mean JADAS27 and CHAQ-DI were approximately twofold higher (ie, worse) throughout the study among patients not receiving concomitant methotrexate versus those receiving methotrexate in the LOCF analyses, whereas mean values were similar in the observed analyses (online supplementary figure 3B).

### Predictor analysis

Early JIA ACR and JADAS27 LDA and ID responses (ie, at weeks 4, 8, 12 and 16) were the only predictors of JADAS27 clinical remission (ie, sustained JADAS27 ID for ≥6 continuous months; figure 4). Achievement of JIA ACR 90 at week 12 was most predictive of later achievement JADAS27 clinical remission (online supplementary figure 4).

## DISCUSSION

This long-term open-label extension study demonstrated that adalimumab was well tolerated and effective among children with polyarticular JIA through 6 years of exposure with or without background methotrexate therapy but was associated with a relatively low treatment retention rate. No new safety signals were identified in this study. Serious AEs and serious infections were within the range of those reported for other pediatric populations treated with adalimumab.^21^ Overall, the adalimumab safety profile in this study was consistent with that of adult populations.^22^ No increased safety risk was observed in patients who were AAA positive versus those who were AAA negative in the presence or absence of methotrexate.

These findings were also consistent with data from the real-world STRIVE registry in patients with active polyarticular JIA.^23 24^ In the 7-year interim analysis of STRIVE, the rate of serious AEs was 7.2/100 PY and the rate of serious infections was 2.0/100 PY with 1855.5 PY of adalimumab exposure (± methotrexate). Similar results were demonstrated in the German BiKeR registry in patients with polyarticular JIA (serious AEs, 11.0/100 PY; serious infections, 5.5/100 PY).^25^

This study enrolled children with JIA who failed previous NSAID treatment or methotrexate monotherapy, and had active disease at baseline. After open-label adalimumab treatment was initiated, clinical responses were achieved as early as week 2 and maintained throughout the study with more than one-third of patients achieving JADAS27 clinical remission (ie, sustained JADAS27 ID ≥6 continuous months) after 4 years of treatment. The results of the conservative NRI analysis, as opposed to the observed analysis, and their interpretation were impacted by the high study discontinuation rate. Although the retention rate of treatment was relatively low, it is in line with other long-term JIA extension studies; 36% of patients completed a 7-year abatacept trial,^26^ 30% completed a 204-week (4-year) infliximab trial^27^ and 38% completed 7 years in an etanercept study.^28^ These low retention rates observed in many JIA trials highlight the need to have alternative therapies or different treatment strategies, such as the treat-to-target, to better achieve long-term disease control.^29^ Of note, in this study, most discontinuations during the extension period were due to withdrawal of consent (n=18), lost to follow-up (n=13) and other (n=19, including one patient in remission) versus AEs (n=6) or lack of efficacy (n=7); however, all discontinuations are counted as lack of response in the NRI analysis to provide a conservative estimate in line with the intention-to-treat principles.

The findings of this study also indicated that early clinical response predicted achievement of JADAS27 clinical remission. In a previous study, JIA ACR 70 response at 4 months was a predictor of disease remission in patients with methotrexate-refractory JIA who were receiving etanercept.^30^ Similarly, a study in patients with polyarticular JIA receiving methotrexate with or without etanercept and prednisolone demonstrated that JIA ACR 70 response at 4 months was a strong predictor of achievement of clinical ID.^31^ Similar results have been demonstrated in rheumatoid arthritis studies in which early response to treatment predicted remission or good clinical response long term.^32–^^34^ Furthermore, a poor response (assessed by JIA ACR 30 and 70) to 6-month treatment with methotrexate in patients with polyarticular JIA was associated with higher disability, antinuclear antibody negativity, and longer disease duration in the PRINTO trial.^35^ An analysis of the BiKeR registry demonstrated that earlier treatment with a biological agent (within 2 years of symptom onset) was associated with better disease control and drug-free remission.^36^ Taken together, these results suggest that early response to treatment is associated with disease remission in patients with JIA, and therapy adjustments are warranted early on in patients who fail to respond. Of note, RF negativity was not a predictor of achieving remission in our analysis, although RF-positive polyarticular JIA has been associated with lower rates of remission than other JIA forms in the past.^37–^^39^

The strengths of this study include long follow-up duration, inclusion of response results for both BSA and FD dosing regimens, and the consistency of efficacy and safety results throughout the years in an established population (polyarticular forms of JIA). Other strengths include using both JIA ACR response criteria and JADAS as efficacy measures to complement each other and measurement of more stringent levels of response (such as JADAS27 ID and JADAS27 clinical remission). The 128 patients who entered the long-term extension had similar baseline characteristics as the overall population, and therefore were representative of the overall patient population. In common with other JIA long-term extension studies, a major limitation was the low retention rate, with many patients discontinuing during the long-term extension period. Additionally, the sample size was not large enough for the evaluation of rare AEs, no radiological data were collected, and there may be a potential dropout bias.

### Conclusions

The results of this study show that long-term adalimumab therapy is well tolerated and effective in patients with polyarticular JIA. However, the low treatment retention rate should be noted when assessing these results. Overall, our results support the use of adalimumab as a therapy option for patients with active polyarticular JIA.

**Key messages**

**What is already known about this subject?**

- Adalimumab has a favourable benefit–risk profile in children with JIA, but extended long-term safety and efficacy outcomes and predictors of sustained disease control have not been previously assessed.

**What does this study add?**

- This study investigated the long-term safety and efficacy of adalimumab, and demonstrated that adalimumab was well tolerated and effective among children with polyarticular JIA through 6 years of exposure with or without background methotrexate therapy.
- The study results also indicated that early clinical response predicted achievement of JADAS27 clinical remission.

**How might this impact on clinical practice or future developments?**

- These results support the long-term use of adalimumab as a therapy option for patients with active polyarticular JIA.
- Furthermore, early response to treatment was shown to be associated with disease remission in patients with JIA, suggesting that therapy adjustments are warranted early in patients who fail to respond.
