# Supplementary material for: Long-term outcomes in patients with polyarticular juvenile idiopathic arthritis receiving adalimumab with or without methotrexate
Source: RMD Open. 2020 Jul 13;6(2):e001208. doi: 10.1136/rmdopen-2020-001208 (PMC7425194; doi:10.1136/rmdopen-2020-001208)

Supplemental Table 1. Incidence of Adverse Events Among Patients Receiving and Not Receiving Methotrexate

AE, n (%)	MTX Use n=85	Non-MTX Use n=86	Patients (N=171)
Any AE	74 (87.1)	71 (82.6)	145 (84.8)
AE possibly drug related	53 (62.4)	55 (64.0)	108 (63.2)
Serious AE	3 (3.5)	5 (5.8)	8 (4.7)
Severe AE	5 (5.9)	5 (5.8)	10 (5.8)
AE leading to discontinuation	2 (2.4)	7 (8.1)	9 (5.3)
Infection	34 (40.0)	38 (44.2)	72 (42.1)
Serious infection	0	2 (2.3)	2 (1.2)
Opportunistic infection*	0	0	0
Malignancy	0	0	0
Injection site reaction	35 (41.2)	37 (43.0)	72 (42.1)
Death	0	0	0
**AEs in ≥5% of patients, n (%)**			
Injection site pain	22 (25.9)	26 (30.2)	48 (28.1)
Injection site reaction	15 (17.6)	12 (14.0)	27 (15.8)
Headache	8 (9.4)	8 (9.3)	16 (9.4)
Upper respiratory tract infection	6 (7.1)	9 (10.5)	15 (8.8)
Contusion	9 (10.6)	5 (5.8)	14 (8.2)
Viral infection	6 (7.1)	7 (8.1)	13 (7.6)
Nausea	6 (7.1)	5 (5.8)	11 (6.4)

Rash	6 (7.1)	5 (5.8)	11 (6.4)
Juvenile arthritis	2 (2.4)	8 (9.3)	10 (5.8)
Hypersensitivity	6 (7.1)	4 (4.7)	10 (5.8)
Arthropod bite	6 (7.1)	4 (4.7)	10 (5.8)
Cough	5 (5.9)	4 (4.7)	9 (5.3)
Oropharyngeal pain	2 (2.4)	7 (8.1)	9 (5.3)

AE, adverse event; MTX, methotrexate.
*Including tuberculosis.

Supplemental Table 2. Overview of Treatment-Emergent AEs by AAA Status in the Open-Label Lead-in Phase

	With methotrexate		Without methotrexate	
**n (%)**	**AAA+ (n=4)**	**AAA- (n=81)**	**AAA+ (n=15)**	**AAA- (n=71)**
AE	4 (100)	70 (86.4)	15 (100)	56 (78.9)
Serious AE	0 (0)	3 (3.7)	1 (6.7)	4 (5.6)
Severe AE	0 (0)	5 (6.2)	1 (6.7)	3 (4.2)
AE leading to discontinuation of study drug	0 (0)	2 (2.5)	1 (6.7)	6 (8.5)
AE at least possibly drug-related	3 (75.0)	50 (61.7)	9 (60.0)	46 (64.8)
Infectious AE	0 (0)	37 (45.7)	6 (40.0)	33 (46.5)
Serious infectious AE	0 (0)	0 (0)	0 (0)	2 (2.8)
AE of malignant neoplasms	0 (0)	0 (0)	0 (0)	0 (0)
Injection site reaction related AE	2 (50.0)	33 (40.7)	6 (40.0)	31 (43.7)
AE of immunologic reaction	0 (0)	7 (8.6)	3 (20.0)	2 (2.8)
AE of opportunistic infection including tuberculosis	0 (0)	0 (0)	0 (0)	0 (0)
Death	0 (0)	0 (0)	0 (0)	0 (0)

AAA, anti-drug antibody; AE, adverse event.

Supplemental Table 3. Overview of Treatment-Emergent AEs by AAA Status and Methotrexate Use in the Double-Blind Period

	With methotrexate n (%)				Without methotrexate n (%)			
	AAA+		AAA-		AAA+		AAA-	
**Patients With:**	**Placebo (n=1)**	**Adalimumab (n=2)**	**Placebo (n=36)**	**Adalimumab (n=36)**	**Placebo (n=1)**	**Adalimumab (n=12)**	**Placebo (n=27)**	**Adalimumab (n=18)**
AE	0 (0)	1 (50.0)	27 (75.0)	31 (86.1)	1 (100)	12 (100)	20 (74.1)	16 (88.9)
Serious AE	0 (0)	0 (0)	2 (5.6)	3 (8.3)	0 (0)	1 (8.3)	0 (0)	0 (0)
Severe AE	0 (0)	0 (0)	0 (0)	2 (5.6)	0 (0)	0 (0)	0 (0)	1 (5.6)
AE leading to discontinuation of study drug	0 (0)	0 (0)	0 (0)	0 (0)	0 (0)	0 (0)	0 (0)	0 (0)
AE at least possibly drug-related	0 (0)	1 (50.0)	15 (41.7)	21 (58.3)	0 (0)	5 (41.7)	9 (33.3)	11 (61.1)
Infectious AE	0 (0)	0 (0)	19 (52.8)	22 (61.1)	0 (0)	8 (66.7)	11 (40.7)	11 (61.1)
Serious infectious AE	0 (0)	0 (0)	0 (0)	1 (2.8)	0 (0)	1 (8.3)	0 (0)	0 (0)
AE of malignant neoplasms	0 (0)	0 (0)	0 (0)	0 (0)	0 (0)	0 (0)	0 (0)	0 (0)
Injection site reaction related AE	0 (0)	1 (50.0)	9 (25.0)	13 (36.1)	0 (0)	3 (25.0)	4 (14.8)	8 (44.4)
AE of immunologic reaction	0 (0)	0 (0)	0 (0)	2 (5.6)	0 (0)	1 (8.3)	0 (0)	2 (11.1)
AE of opportunistic infection including tuberculosis	0 (0)	0 (0)	0 (0)	0 (0)	0 (0)	0 (0)	0 (0)	0 (0)
Death	0 (0)	0 (0)	0 (0)	0 (0)	0 (0)	0 (0)	0 (0)	0 (0)

AAA, anti-drug antibody; AE, adverse event.

Supplemental Figure 1. Study schematic. ADA, adalimumab; BSA, body surface area; eow, every other week; MTX, methotrexate.

*BSA dosing eow for 44–136 weeks. †Fixed dosing (<30 kg: 20 mg eow; ≥30 kg: 40 mg eow) thereafter.

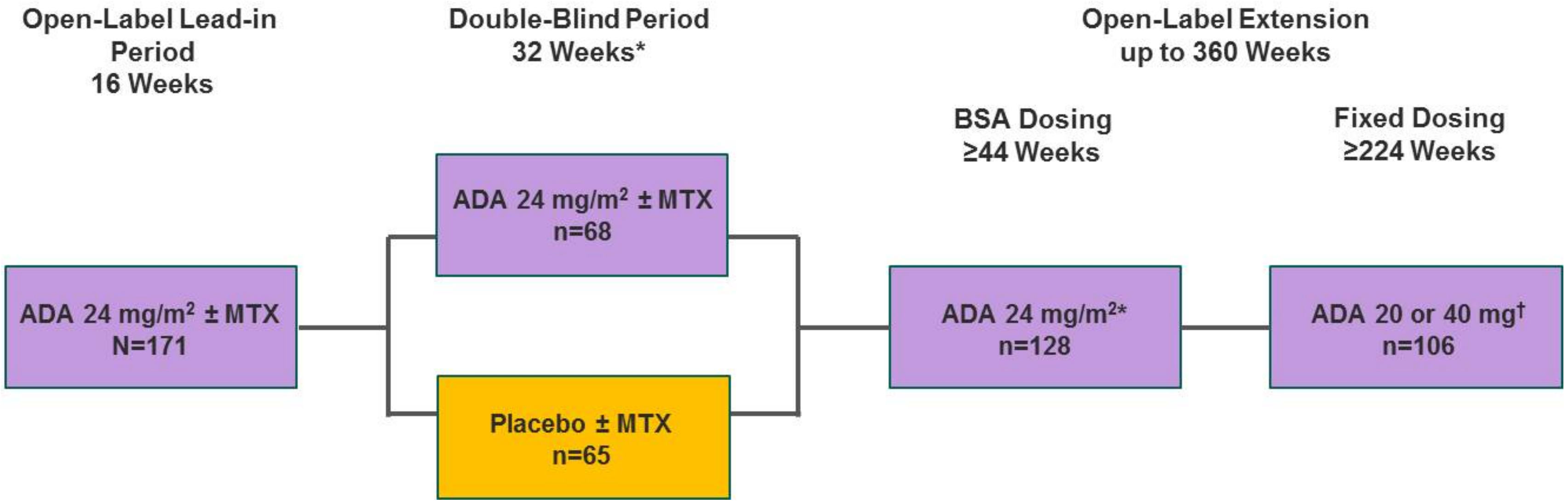

Supplemental Figure 2. Percentage of Patients Achieving JIA ACR 30/50/70/90 Over Time Stratified by Concomitant MTX Use. JIA ACR 30/50/70/90, 30%, 50%, 70%, or 90% improvement in the Pediatric American College of Rheumatology response; MTX, methotrexate; NRI, non-responder imputation. Closed symbols=observed analysis. Open symbols=NRI analysis. For observed analysis, n values are shown for weeks 0, 104, 200, and 312; for NRI analysis, N=85 (MTX) and N=86 (non-MTX).

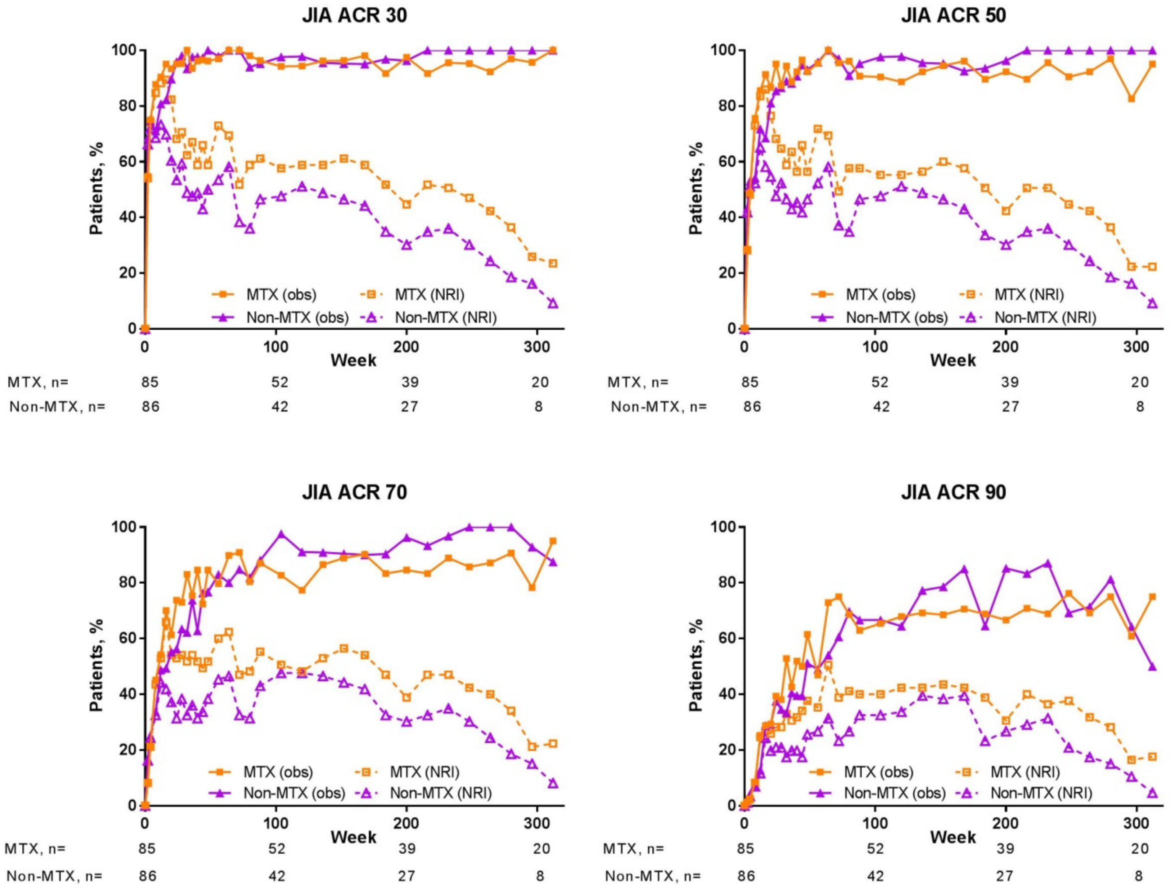

Supplemental Figure 3. Percentage of Patients Achieving (A) JADAS27 LDA and JADAS27 Inactive Disease and (B) Mean JADAS27 LDA and CHAQ-DI Over Time Stratified by MTX Use. Closed symbols=observed analysis. Open symbols=NRI and LOCF analyses. CHAQ-DI, Childhood Health Assessment Questionnaire Disability Index; ID, inactive disease; JADAS27, 27-joint Juvenile Arthritis Disease Activity Score; LDA, low disease activity; LOCF, last observation carried forward; MTX, methotrexate; NRI, non-responder imputation. For observed analysis, n values are shown for weeks 0, 104, 200, and 312; for NRI and LOCF analyses, N=85 (MTX) and N=86 (non-MTX). In panel B mean JADAS27 panel, dotted lines represent JADAS27 LDA (≤3.8) and JADAS ID (≤1) cutoffs.

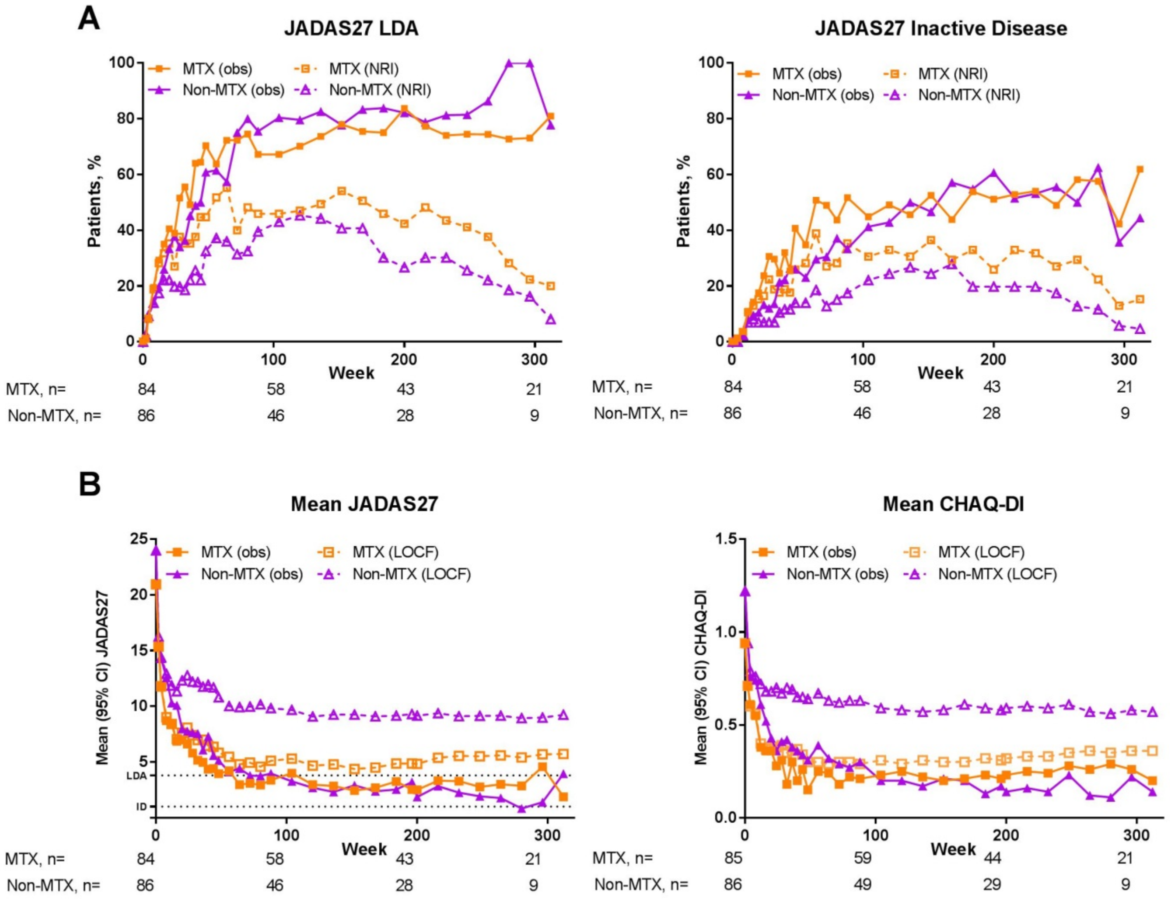

Supplemental Figure 4. Percentage of patients achieving (A) JADAS27 clinical remission over time and (B) regression tree analysis selecting JIA ACR 90 response at week 12 as a significant factor associated with JADAS27 clinical remission. JIA ACR 90, 90% improvement in the Pediatric American College of Rheumatology response. JADAS27, 27-joint Juvenile Arthritis Disease Activity Score; NRI, non-responder imputation. Regression tree analysis: 10 patients with missing JIA ACR 90 status at week 12 were excluded. For observed analysis, n values are shown for weeks 28, 104, 200, and 312; for NRI analysis, N=171.

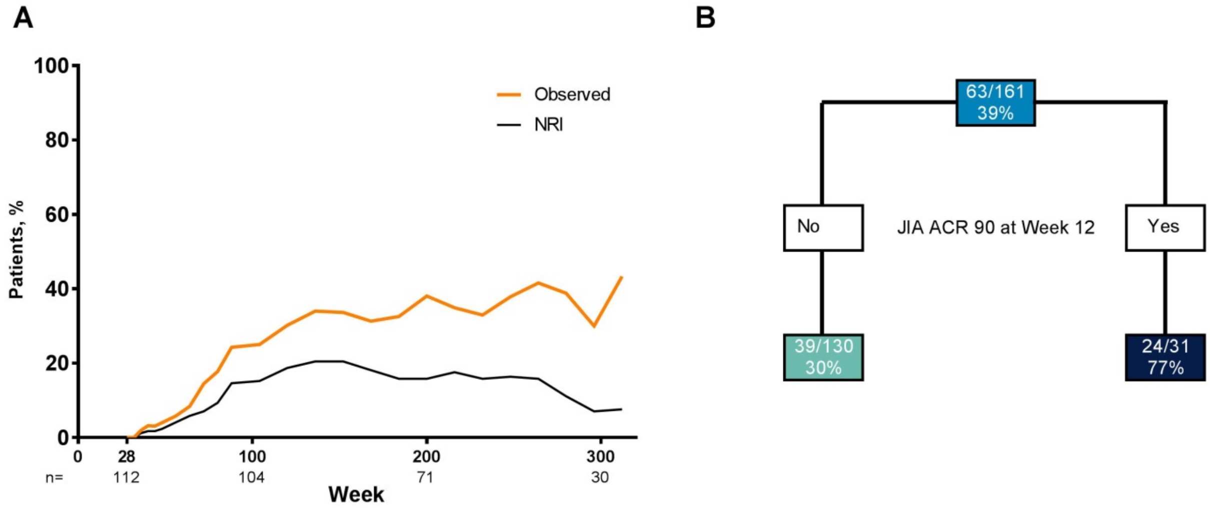

Supplement: Supplementary data [file rmdopen-2020-001208s022.pdf]
